# Directed evolution of super-secreted variants from phage-displayed human Interleukin-2

**DOI:** 10.1038/s41598-018-37280-5

**Published:** 2019-01-28

**Authors:** Gertrudis Rojas, Tania Carmenate, Julio Felipe Santo-Tomás, Pedro A. Valiente, Marlies Becker, Annia Pérez-Riverón, Yaima Tundidor, Yaquelín Ortiz, Jorge Fernandez de Cossio-Diaz, Luis Graça, Stefan Dübel, Kalet León

**Affiliations:** 1 Center of Molecular Immunology, calle 216 esq 15, apartado 16040, Atabey, Playa, CP 11300 La Habana Cuba; 2 Biology Faculty, La Habana University, 25 e/ I y J, Vedado, Plaza, CP 10400 La Habana Cuba; 3 Technische Universität Braunschweig, Institute of Biochemistry, Biotechnology and Bioinformatics, Department of Biotechnology, Spielmannstraße 7, 38106 Braunschweig, Germany; 4 Instituto de Medicina Molecular, Faculdade de Medicina da Universidade de Lisboa, Lisbon, Portugal

## Abstract

Selection from a phage display library derived from human Interleukin-2 (IL-2) yielded mutated variants with greatly enhanced display levels of the functional cytokine on filamentous phages. Introduction of a single amino acid replacement selected that way (K35E) increased the secretion levels of IL-2-containing fusion proteins from human transfected host cells up to 20-fold. Super-secreted (K35E) IL-2/Fc is biologically active *in vitro* and *in vivo*, has anti-tumor activity and exhibits a remarkable reduction in its aggregation propensity- the major manufacturability issue limiting IL-2 usefulness up to now. Improvement of secretion was also shown for a panel of IL-2-engineered variants with altered receptor binding properties, including a selective agonist and a super agonist that kept their unique properties. Our findings will improve developability of the growing family of IL-2-derived immunotherapeutic agents and could have a broader impact on the engineering of structurally related four-alpha-helix bundle cytokines.

## Introduction

Interleukin-2 (IL-2), originally described as a T-cell growth factor^1^, is currently known to play a pivotal role in immune biology, controlling the delicate balance between tolerance and responsiveness^2^. Structural knowledge about its interactions with the multi-chain IL-2 receptor (IL-2R) contributed to understanding the molecular bases of such a dichotomy^3,4^. Selective blockade of binding interfaces with monoclonal antibodies (mAbs) revealed that IL-2 can enhance effector responses, but also exert a strong down modulatory effect through the expansion of T regulatory cells (Tregs)^5^. After a long history of therapeutic use to promote anti-tumor responses^6^, the functional complexity of IL-2 has resulted in different strategies to treat either cancer or autoimmune/inflammatory conditions^7,8^.

These approaches go beyond the use of the natural cytokine. Disrupting or reinforcing the interactions with particular receptor subunits can segregate the dual roles of the molecule, giving rise to novel therapeutic agents. Impairing IL-2 ability to interact with CD25- the alpha receptor subunit highly expressed on Tregs -by either chemical^9^ or genetic^10^ modification results in potent antitumor activity without the drawbacks of Treg-mediated immune suppression. A “superkine” with increased affinity for CD122 -the beta receptor subunit constitutively expressed in NK and T cells- also has an enhanced effector potential^11^. A variety of IL-2-based antagonists have been generated by disrupting its signalling ability^12–14^. IL-2 engineering does not only target receptor interactions, as fusion to antibody variable and/or Fc domains also modulates its pharmacological properties through changes in bio distribution and plasma half-life, and acquisition of effector functions^15–19^.

Production of recombinant human IL-2 (rhIL-2) and related molecules often relies on cytoplasmic expression in *E. coli* and *in vitro* refolding^20,21^. Refolding procedures have a variable efficiency and need a careful optimization. Alternative expression systems, based on yeast, insect and mammalian cells have also been explored^22^. Regardless of the expression system, IL-2 and other structurally related alpha-helical cytokines have a very strong aggregation propensity, resulting in the formation of aggregates of up to 30 molecules even in optimized formulations^23,24^. A link between aggregation, toxicity and undesired immunogenicity of IL-2 has been postulated to compromise its therapeutic usefulness^25,26^.

Although the initial reports of phage-displayed biologically active IL-2 were published more than 20 years ago^27,28^, this platform has only recently been exploited to map the interactions of hundreds of IL-2-derived variants^29–31^, and IL-2 engineering has been dominated by yeast display^11,32^. Here we report directed molecular evolution of phage-displayed IL-2 resulting in the discovery of single mutations that increase display levels, enhance secretion by human host cells and diminish IL-2 aggregation. The general effect of the identified changes on totally different secretion systems and diverse IL-2-derived molecules is expected to improve the developability potential of the growing family of IL-2-related immunomodulatory agents and opens new avenues for cytokine engineering.

## Results

### Different mutations were found after selection of phage-displayed IL-2 variants on CD25

Selection from a phage library of 10^9^ members displaying hIL-2 with controlled diversity at the IL-2R alpha subunit interface (see Supplementary Table S1 for library design) on immobilized human CD25 rendered phage mixtures with growing reactivity to the selector molecule (Supplementary Fig. S1a). Sequencing of a sample of 30 clones from the fourth panning round output revealed the presence of clones displaying wild-type (wt) hIL-2 (23%), variants with several mutations in the segment 61–74 (mainly V69A/Q74P, 67%), and single-mutated variants (K35E or K35Q,10%). Selection on mouse CD25 (Supplementary Fig. S1b) only rendered recurrent changes at position 35 (K35E, K35Q, or K35D, 13% of 30 additional clones from this panning).

As V69A/Q74P increase the affinity of hIL-2 towards human CD25^32^ and not to its mouse counterpart^12^, the appearance of changes in the region 61–74 was readily understood. Mutations at position 35 were unexpected because theoretical diversity at this position only included the original K and the conservative replacement K35R due to the postulated involvement of this residue in forming ionic bonds with CD25, being any other change the consequence of library construction errors (during mutagenic oligonucleotide synthesis or DNA polymerization). A sample of clones (30) from the unselected library (all able to display hIL-2 as judged by reactivity with anti-tag Myc1–9E10 mAb recognizing the *c-myc* tag fused to the displayed proteins) was used to evaluate any possible bias in the original library diversity at position 35. While the presence of two clones with undesired changes at position 35 (K35T) provided actual evidence for the existence of library construction errors, most clones had sequences corresponding to the theoretical library design, even at that position, ruling out gross library construction mistakes. Over-representation of E, Q and D at position 35 was not found, seeming to be an actual selection-driven feature.

Even knowing that the library contains errors and these undesired changes could in principle be selected if they confer a binding advantage to the selector target, the emergence of charge reversal mutations at position 35 upon selection on CD25 was surprising. The positively charged K35 has been postulated to form an ionic bond with E1 from the human alpha IL-2R subunit, on the basis of the known crystal structure of the complex^4^. Non-conservative replacements at this position are thus supposed to result in a weaker interaction with CD25, not the other way around. Further experiments were then aimed at deciphering the driving forces behind this enrichment.

### Mutations at position 35 improve the display of IL-2 on filamentous phages

**Figure 1:**
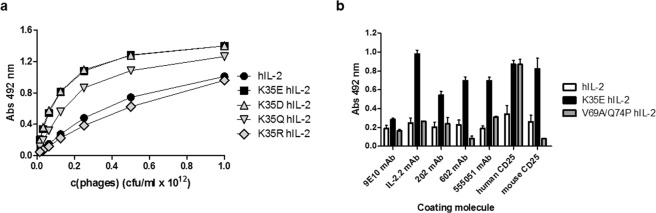
Effect of mutations at position 35 on IL-2-derived proteins displayed on filamentous phages. Display levels of mutated hIL-2 variants were evaluated by ELISA (**a**). Equivalent amounts of phage particles (as determined by viral titration) were incubated on polyvinyl chloride microplates coated with the anti-*c-myc*-tag mAb Myc1-9E10 that recognizes proteins fused to PIII. Bound phages were detected with an anti-M13 mAb conjugated to horseradish peroxidase. Phages displaying non-mutated hIL-2 were used as reference. The quality of the displayed proteins was evaluated in a second ELISA (**b**). Phages carrying either non-mutated hIL-2, or hIL-2 containing K35E replacement or the double mutation V69A/Q74P, were diluted to reach similar levels of the displayed proteins (according to Myc1-9E10 reactivity in the previous experiment) and incubated on microplates coated with several anti-IL-2 monoclonal antibodies and CD25 (human/mouse). Bound phages were detected as described in (**a**). Parallel production and ELISA evaluation of phages displaying each variant were independently repeated three times to assess reproducibility of the differences. The evaluation of a representative phage production experiment is shown in each panel. Samples were evaluated by triplicate on ELISA plates.

Replacements at position 35 resulted in a remarkable increase in the display levels of hIL-2, as determined by phage enzyme-linked immunosorbent assay (ELISA) on immobilized Myc1–9E10 mAb (Fig. 1a). Charge inversion replacements (K35E, K35D) had higher influence than K35Q, while the conservative substitution K35R had no effect. K35E -the mutation most frequently selected during panning- was chosen for further experiments. Display levels of a variety of IL-2-derived muteins already described^10,11,14,32^ containing K35E were also increased by 6-30-fold. K35E not only improved the levels, but also increased the antigenicity (measured with several anti-IL-2 mAbs) and receptor binding ability (to human and mouse CD25) to different extents (Fig. 1b). This was in sharp contrast to the specific increase in binding to human CD25 observed for the already described V69A/Q74P IL-2R alpha subunit super-binder^32^. The improved binding to multiple partners recognizing different epitopes of native hIL-2, particularly to IL-2.2 mAb that recognizes the segment E99-M104 located far from the region targeted in the library^29^, provided a first indication that the observed improvement is a result of a global effect of K35E on folding of the displayed protein.

### The effect of K35E extends to secretion of IL-2-containing fusion proteins by mammalian cells

**Figure 2:**
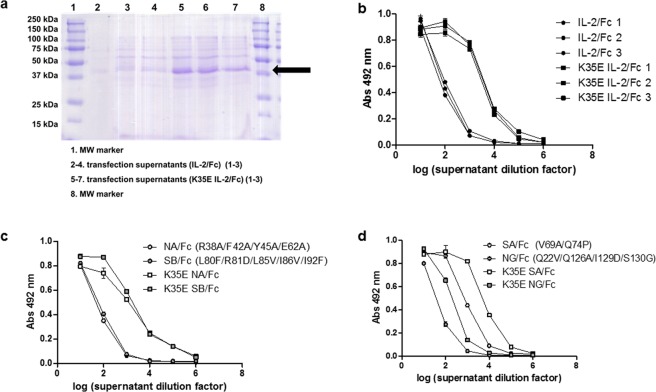
Effect of K35E mutation on the secretion of IL-2-derived recombinant proteins by human cells. The presence of Fc-fused IL-2 (either non-mutated or containing K35E) in transiently transfected Expi293 supernatants was evaluated by SDS-PAGE under reducing conditions (**a**). Three independent transfections were performed for each protein variant to assess reproducibility. The arrow indicates the band corresponding to monomeric fusion protein. The result was confirmed by ELISA on microplates coated with the anti-IL-2 mAb IL-2.2 (**b**). Bound fusion proteins were detected with an anti-human Fc antibody conjugated to horseradish peroxidase. A similar comparison was done for fusion proteins containing four reported IL-2-derived muteins: an IL-2 receptor alpha subunit non-binder^10^ (NA, **c**), a beta subunit super-binder^11^ (SB, **c**) an alpha receptor super-binder^29^ (SA, **d**) and a gamma non-binder (NG, **d**). The ELISA evaluation of a single representative cell transfection experiment is shown in (**c**,**d**). Samples were evaluated by triplicate on ELISA plates.

The introduction of K35E resulted in an enhanced secretion of IL-2/human Fc fusion proteins after transient transfection of Expi293 cells. This was shown by both SDS/PAGE under reducing conditions and ELISA. The appearance of an intense band with the expected electrophoretic mobility for the IL-2 K35E/Fc fusion monomer in SDS/PAGE of cell transfection supernatants (not observed for the non-mutated fusion protein) indicates higher expression of such mutated version as compared to its wt counterpart (Fig. 2a), which was confirmed by sandwich ELISA with specific antibodies to the IL-2 and Fc moieties (Fig. 2b). Noteworthy, the increase in the intensity of all the other bands coming from cell debris in IL-2 K35E/Fc-containing supernatants reflects a moderately higher growth of cultures expressing the mutated protein.

**Figure 3:**
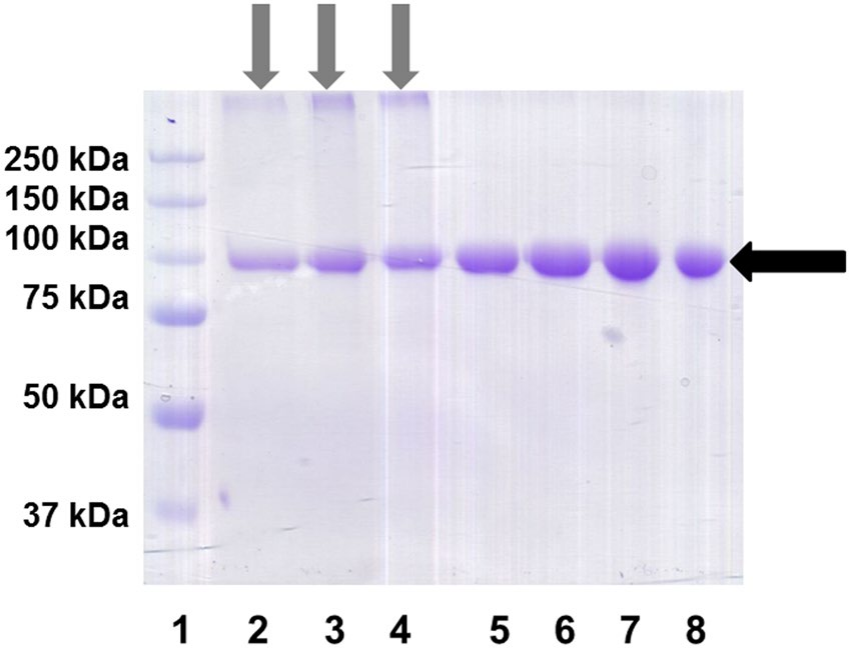
Purified recombinant fusion proteins (after transient Expi293 transfection, protein A affinity chromatography and desalting) were analysed by SDS/PAGE under non-reducing conditions. Five µg of protein were applied to each lane. Several independent batches of each protein were tested (IL-2/Fc in lanes 2–4 and K35E IL-2/Fc in lanes 5–8). Black arrow indicates the major band corresponding to the fusion protein homodimer. Grey arrows highlight the presence of denaturation-resistant aggregates with lower electrophoretic mobility in all samples of non-mutated IL-2/Fc.

The average yield of protein A-affinity purified final product (calculated using seven independent cell transfection experiments) was 8.7 ± 2.2 mg of the recombinant protein from 1 L of culture for the non-mutated IL-2/Fc. This number increased to 92.7 ± 17.5 mg/L for its K35E counterpart. Table 1 shows the individual yields for each batch of purified protein. Characterization of the purified recombinant proteins by SDS/PAGE under non-reducing conditions showed the presence of a single band corresponding to the IL-2 K35E/Fc homodimer (Fig. 3). A similar major band was observed for the IL-2/Fc protein, but in this case it was always accompanied by lower mobility bands corresponding to denaturation-resistant large aggregates that disappeared in reducing conditions (data not shown) and were interpreted as a first indication of a change in the biophysical properties of K35E-mutated IL-2.

**Table 1:** Yield (mg of Protein A-purified recombinant fusion protein from 1 L of culture).

Batch	IL-2/Fc	IL-2 (K35E)/Fc
1	10	106
2	12	78
3	9	115
4	6	71
5	6	80
6	10	110
7	8	89
Mean ± SD (7 batches)	8.7 ± 2.2	92.7 ± 17.5

Secretion-enhancing effects were also shown for fusion proteins including four IL-2-derived muteins (Fig. 2c,d), despite their different basal expression levels, and were observed in different host cells (HEK293-6E and HEK293-T adapted to grow in suspension). Secretion was increased in the whole set of experiments ranging from three to 24-fold. As the secretion machineries of *E. coli* (used for phage display) and human cells are quite different, similar effects in both systems suggest that K35E is inducing an intrinsic change of IL-2 folding pathway and/or structure, which facilitates the secretion process.

### The mutation improves not only quantity, but also quality of the secreted proteins

**Figure 4:**
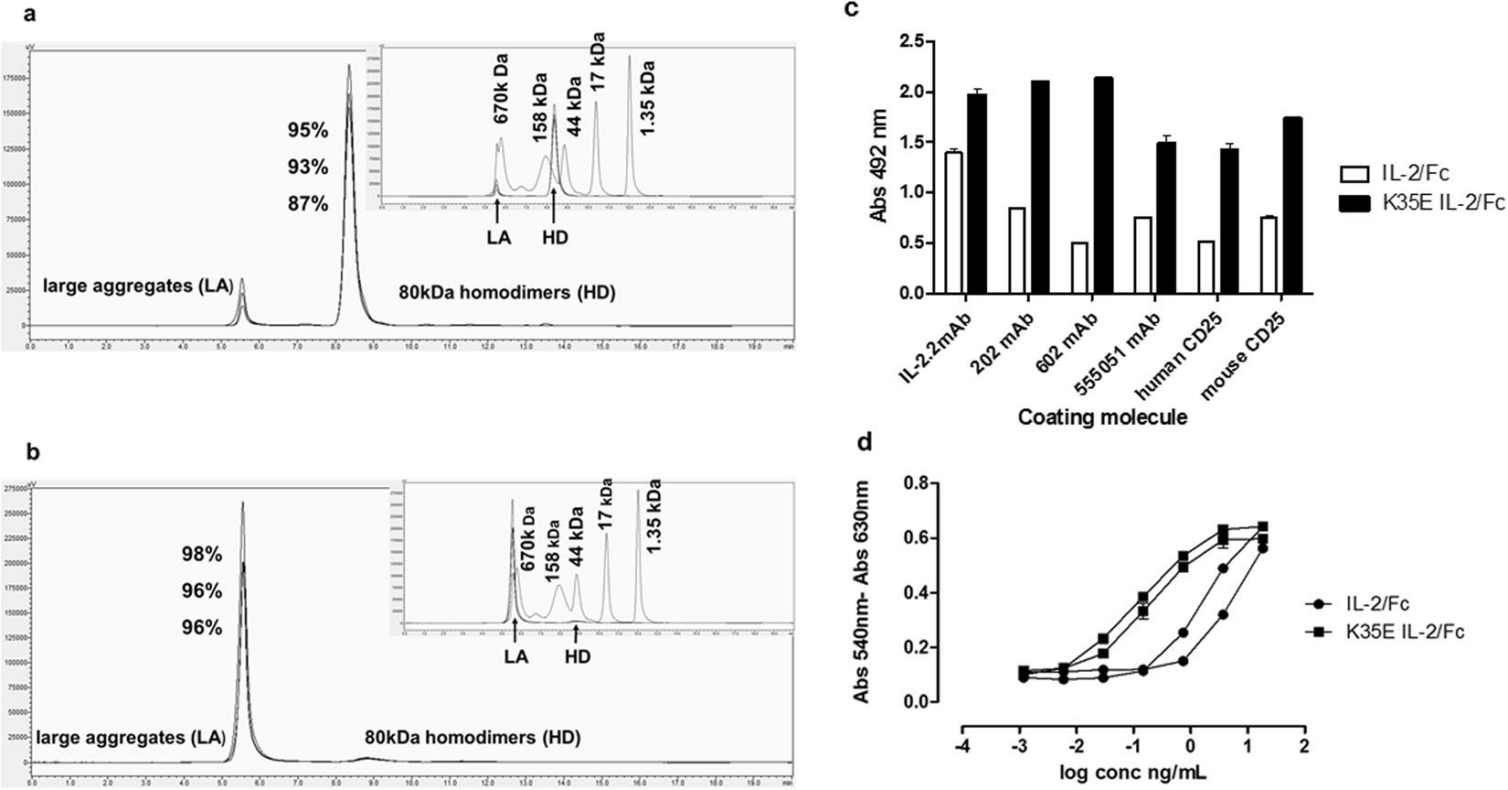
Differences between IL-2/Fc and K35E IL-2/Fc fusion proteins. The aggregation status of purified fusion proteins was compared by size exclusion chromatography (**a**,**b**). The elution profiles of three independent batches of K35E IL-2/Fc (**a**) and non-mutated IL-2/Fc (**b**) are shown. A standard comprising five molecules of known molecular weight (thyroglobulin 670 kDa, IgG 158 kDa, ovalbumin 44 kDa, myoglobin 17 kDa, vitamin B12 1.35 kDa) was used for calibration (overlapping between the elution profiles of samples and standards is shown in the inserts). The numbers indicate the relative areas (%) of the main peaks corresponding to homodimers and large aggregates respectively in each kind of sample. The antigenicity and receptor binding properties of IL-2/Fc and K35E IL-2/Fc were compared by ELISA (**c**) on microplates coated with several anti-IL-2 mAbs and CD25 (human/mouse). Bound proteins were detected with an anti-human Fc antibody conjugated to horseradish peroxidase. The ELISA evaluation of one batch of each fusion protein (both produced in parallel) is shown as a representative example. Samples were evaluated by triplicate in ELISA. The result was confirmed in independent ELISAs with the remaining two batches of each. The ability of two batches of each fusion protein to induce the proliferation of the IL-2-dependent CTLL-2 cell line was evaluated (**d**), using the Alamar blue dye reduction as an indicator of cell proliferation.

The aggregation propensity of the recombinant fusion proteins was finally investigated through size exclusion chromatography. The mutated K35E IL-2/Fc protein exhibited a much lower aggregation propensity than its non-mutated counterpart. While purified K35E IL-2/Fc predominantly exists as a homodimer (Fig. 4a) due to the presence of the dimerizing Fc moiety, IL-2/Fc tends to form higher molecular weight aggregates (Fig. 4b). This finding provided an initial clue to the mechanism behind the already observed secretion increase, as aggregation could compete with normal transit through the secretion pathway precluding high level production of recombinant proteins from transfected cells. Expression of an aggregation-prone recombinant protein can also compromise cell viability, contributing to the final effect through a decrease in the number of cells able to produce it.

K35E IL-2/Fc homodimers kept IL-2 antigenicity and receptor binding properties, as shown by recognition by four anti-IL-2 mAbs and human/mouse CD25 (Fig. 4c). K35E IL-2/Fc was always more reactive than the original IL-2/Fc, indicating that K35E replacement gives rise to a protein that reproduces the global structure of natural IL-2 to a greater extent than its non-mutated recombinant counterpart. Heavy aggregation of the latter can hide IL-2 epitopes, explaining the diminished reactivity towards both antibodies and receptors. These findings resemble the results with the phage-displayed mutated protein (see above).

K35E IL-2/Fc also induced proliferation of the IL-2-dependent CTLL-2 cell line *in vitro* (Fig. 4d), showing compatibility of K35E with cell signalling. The specific activity of twelve batches of K35E IL-2/Fc was in the expected range (2.15 × 10^6^ to 1.4 × 10^7^ IU/mg) in that assay, which is the standard technique to quantitate the biological activity of the cytokine^33^. The aggregation status of the non-mutated IL-2/Fc was reflected in a lower specific activity (1.2–5 × 10^5^ IU/mg), confirming the higher quality of the mutated version.

Even though the higher receptor binding ability and biological activity of the mutated protein could be interpreted as the result of a specific increase in the affinity to CD25 (the selector target used for the initial screening), two observations argue against this alternative explanation: i) the proximity of the positively charged group of K35 to the negatively charged E1 of CD25 in the cytokine/receptor complex^4^ that makes it very unlikely an affinity improvement due to the replacement K35E, and ii) the general enhancing effect of K35E on binding to other molecules like antibodies recognizing a plethora of different IL-2 epitopes (not used for phage selection). The large differences between the aggregation status of wt and mutated IL-2-containing fusion proteins has precluded a strict comparison of their binding affinities to definitely rule out a contribution of intrinsic receptor affinity.

### K35E induces a bend in the *N*-terminal helix of IL-2 and increases the stability and solubility of the molecule

**Figure 5:**
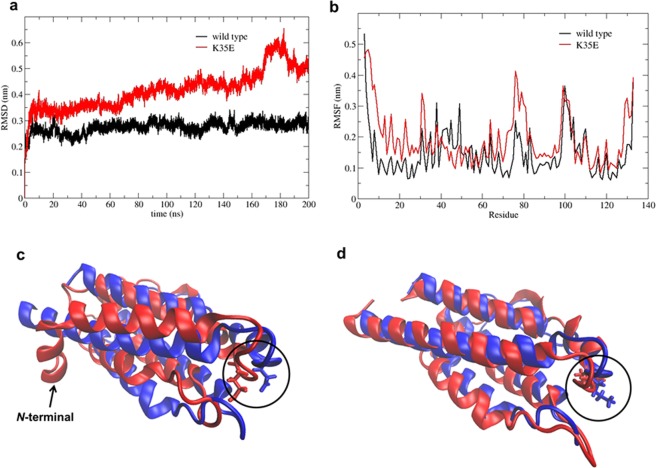
*In silico* study of the impact of K35E over the structural stability of human IL-2. Root mean square deviation (RMSD) of heavy atoms of IL-2 (black line) and K35E variant (red line) over a 200 ns molecular dynamics (MD) simulation are shown in (**a**). Root mean square fluctuation (RMSF) per residue along the primary sequence of IL-2 (black line) and K35E variant (red line) are shown in (**b**). Panels c and d show the structural superposition of the initial (0 ns, blue) and final (200 ns, red) structures from the MD simulations using the mutated and non-mutated proteins respectively. The arrow indicates the bending on the *N*-terminal segment in the K35E-containing variant, while the circle encloses the replacement at position 35.

A hypothesis to explain the observed differences was generated from *in silico* molecular dynamics (MD) simulations of the wt and K35E variants. Whereas stable RMSD values below 0.3 nm were observed for wt IL-2, the time profile of the RMSD values calculated for heavy atoms of the mutated protein with respect to the initial structure (>0.4 nm) indicated the occurrence of conformational changes over the simulation (Fig. 5a). The flexibility differences between both proteins were dissected by calculating the root means square fluctuation (RMSF) per residue. The *N*-terminal segment of the mutated protein showed more structure fluctuation compared to the wt (Fig. 5b). The structural superposition of the initial and final structures of the K35E variant simulation showed a distinctive bend in the *N*-terminal helix (Fig. 5c) not observed for non-mutated IL-2 (Fig. 5d), indicating a long-distance effect of the mutation on the IL-2 structure.

Calculation of the difference in the folding free energy between the mutated and the wt protein (ΔΔG_folding_ = ΔG _folding wt_ − ΔG _folding K35E_) according to the thermodynamic cycle shown in Supplementary Fig. S2 rendered a positive ΔΔG_folding_ value (17.49 kcal/mol), indicating that K35E behaves as a stabilizing mutation. Solubility was also studied *in silico*, through calculation of the difference in the solvation free energy of the folded state of the mutated versus the wt protein (ΔG_solvation_ = G_solvation wt_ − G_solvation K35E_). A positive ΔG_solvation_ value (23.94 kcal/mol) was consistent with the lower aggregation propensity of the mutated protein.

The limited availability and high aggregation-propensity of non-mutated IL-2-containing fusion proteins have hampered verification of the structural and biophysical effects of K35E up to now. Only experimental studies could confirm the individual contribution of flexibility, conformational changes and thermal stability to the improved secretion of the mutated products.

### Super-secreted K35E IL-2 fusion protein has immunostimulatory and anti-tumoral activity *in vivo*

The low yields of non-mutated IL-2/Fc, together with its lower specific activity compared to the K35E-containing version, precluded a direct comparison between both molecules in an *in vivo* scenario. Using equal masses of them would give a strong advantage to the more active mutated product, while pairing the amounts to reach the same levels of biological activity (according to the above described CTLL-2 proliferation assay that is commonly used for that purpose), would have required large quantities of the non-mutated less active product. Production of such amounts was not affordable due to the intrinsic low yields of this protein. The different aggregation status of both proteins (see above) would complicate further any comparison of *in vivo* behaviour. Therefore, our analysis of the *in vivo* function of K35E IL-2/Fc in C57/BL6 mice was only aimed at determining whether the mutated highly secreted product exhibits the pleiotropic functions already described for Il-2 or not.

**Figure 6:**
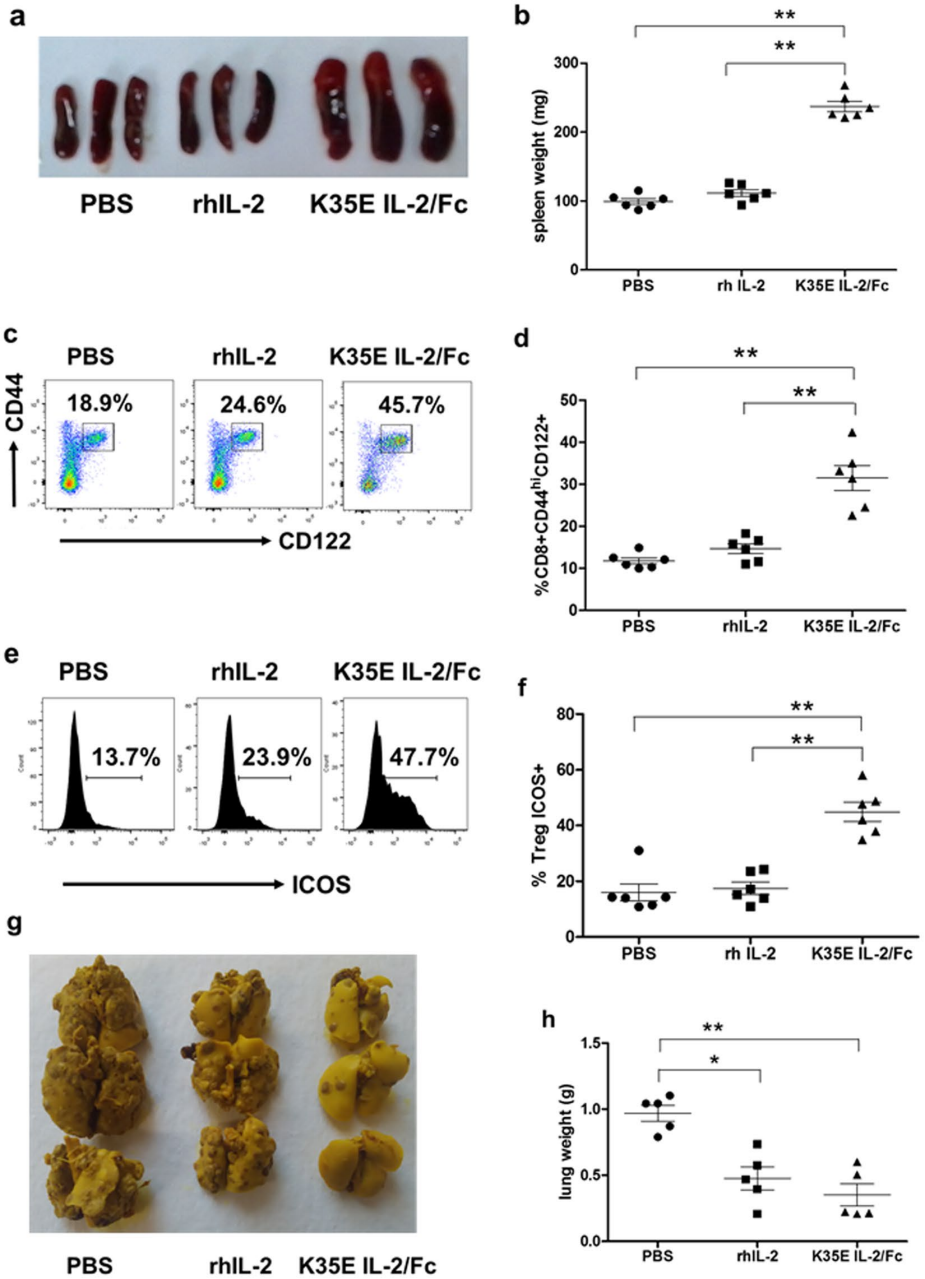
*In vivo* functional characterization of K35E IL-2/Fc protein. C57/BL6 mice received five daily injections of either rhIL-2 or K35E IL-2/Fc (4 × 10^4^ IU) and were sacrificed on day 7. Control mice were injected with PBS. Spleen enlargement is shown in the picture (**a**) and spleen weights are represented in (**b**). The expansion of memory phenotype CD8 + CD44^hi^CD122+ lymphocytes (**c**,**d**) and Treg CD4 + CD25 + Foxp3 + ICOS + population (**e**,**f**) in the spleens of treated mice was assessed by flow cytometry. The antimetastatic effect was evaluated 21 days after the inoculation of MB16F0 melanoma cells, followed by the above described treatments (with rhIL-2, K35E IL-2/Fc or PBS). Representative lungs are shown in (**g**), and the lung weights of the whole set of animals are represented in (**h**). Lines represent mean and standard deviation within each group. Asterisks stand for statistical significance (*p < 0.05, **p < 0.01). Bonferroni test was used for multiple comparisons. All the experiments were performed twice.

K35E IL-2/Fc was strongly immunostimulatory *in vivo*, as judged by the enlargement of the mice spleens (Fig. 6a,b). This effect was associated to the expansion of both CD44^hi^ memory-phenotype CD8+ T cells (Fig. 6c,d) and the highly suppressive ICOS+ subpopulation of CD4 + CD25 + Foxp3 + Tregs^34^ (Fig. 6e,f). These results showed the compatibility of K35E with the dual biological roles of IL-2 within the immune system, promoting both effector and regulatory functions. The enhancement of immune responsiveness induced by K35E IL-2/Fc translated into a strong anti-metastatic effect in a mouse model of experimental lung metastasis after injection of MB16F0 melanoma cells (Fig. 6g,h).

Recombinant human IL-2 (rhIL-2, 15 kDa) produced in *E. coli* and refolded *in vitro* was included in all the *in vivo* experiments as an additional control because it is a well-characterized protein commonly used in our laboratory for this kind of experiment and known to have anti-tumor activity^10^, but its effects are not directly comparable to the ones exerted by the fusion protein. The latter has a longer half-life due to its higher molecular weight (80 kDa) and the presence of an Fc moiety able to recirculate through binding to the neonatal Fc receptor. Such a difference explains the more pronounced effects of K35E IL-2/Fc in the *in vivo* scenario, where persistence of the stimulation is a critical factor. The whole set of *in vivo* experiments showed that the introduction of K35E is compatible with immunostimulatory and anti-tumor activity of IL-2-derived molecules.

### Enhanced secretion is compatible with the selective modulation of the interactions with receptor subunits

**Figure 7:**
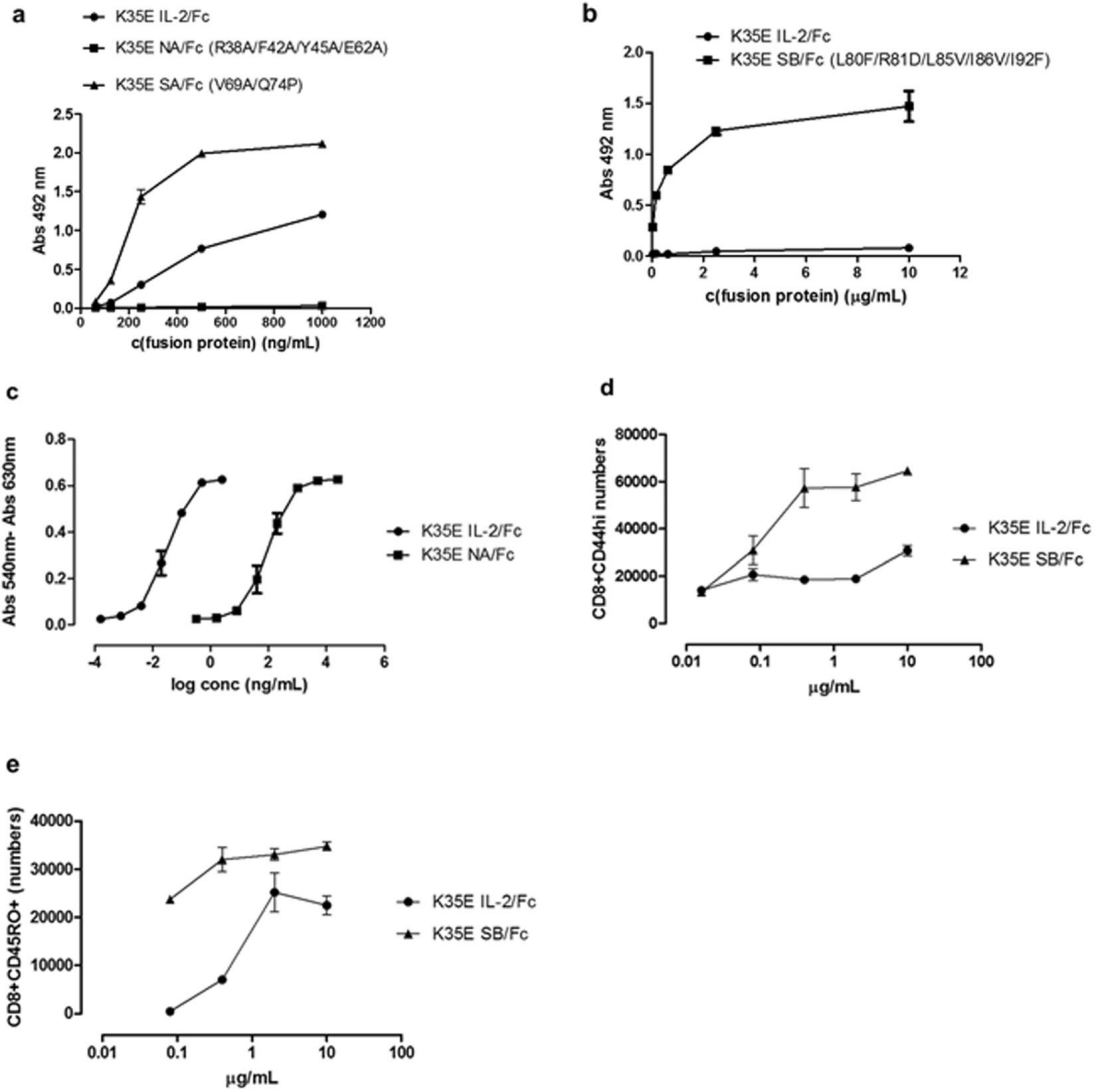
Compatibility between K35E and other mutations aimed at selectively modulating IL-2 interactions with IL-2R subunits. Binding properties of different Fc-fusion proteins were evaluated by ELISA on microplates coated with either human CD25 (**a**) or human CD122 (**b**). In the first experiment (**a**) fusion proteins containing IL-2, an alpha non-binder mutated variant^10^ (NA), or the alpha super-binder named here SA^32^ (all having the additional K35E replacement) were compared. A comparison between fusion proteins containing IL-2 and the beta subunit super-binder SB^11^ (both including K35E) was performed in the second experiment (**b**). Bound proteins were detected with an anti-human Fc antibody conjugated to horseradish peroxidase. ELISA experiments were repeated three times with independent batches of each recombinant protein. The results of the evaluation of one batch of each one are shown as a representative example. Samples were evaluated by triplicate in ELISA. K35E NA/Fc exhibited decreased ability to induce the proliferation of IL-2-dependent CTLL-2 cells as compared to its K35E IL-2 counterpart, as determined with an Alamar blue-based colorimetric assay (**c**). K35E SB/Fc showed an increased capacity to expand memory phenotype CD8+ T lymphocytes of both mouse (**d**) and human (**e**) origin *in vitro*.

The addition of K35E did not impair the effects of sets of mutations aimed at increasing IL-2 reactivity to either alpha^32^ or beta^11^ receptor subunits or selectively disrupting the interaction with the alpha subunit^10^ (Fig. 7a,b). The unique functional properties of two of these variants (the alpha non-binder called no-alpha (NA) and the beta super-binder H9 (named here SB) were preserved after the introduction of K35E in the Fc-fusion format. K35E NA/Fc ability to stimulate CTLL-2 cells having the high affinity trimeric IL-2R was severely reduced (by more than two orders of magnitude) compared to K35E IL-2/Fc (Fig. 7c). While the specific activity of the latter was in the 10^6^–10^7^ IU/mg range as previously described, biological activity of K35E NA/Fc only reached 1–6 × 10^4^ IU/mg, reflecting the strong influence of alpha subunit binding and recapitulating previous findings with the NA molecule produced in *E. coli*^10^.

On the other hand, K35E SB/Fc was a strong super agonist overstimulating the expansion of memory phenotype CD8 + CD44^hi^ T cells in cultures of purified mouse CD8+ T cells (Fig. 7d), as well as the *in vitro* expansion of human CD8 + CD45R0 + T cells (human memory phenotype) (Fig. 7e). This reflected the intrinsic advantage of the SB variant having increased affinity to the IL-2R beta subunit to stimulate effector cells harbouring the dimeric beta/gamma receptor^11^.

## Discussion

Directed evolution by phage display allows mimicking natural selection *in vitro*. New molecules arise from genetically diverse libraries, according to their relative “fitness” in a selection environment dictated by the researcher^35^. While every selection depends on binding to a selector target, “fitness’‘ means more than affinity between library molecules and this target. Other features modulate the likelihood of interactions: expression levels of library genes, compatibility to periplasmic secretion and assembly on phage, folding, aggregation propensity and stability of the displayed proteins, and competition among library members. In the current case, library construction by Kunkel mutagenesis^36^ on an intact IL-2 gene template implied a strong competitive disadvantage of variants mutated at position 35 because of the abundance of native functional IL-2, the presence of molecules with increased affinity for CD25, and the introduction of additional changes with detrimental effects on binding when mutating segment 35–45. Recurrent selection of such variants despite all these counter-selective factors highlighted the importance of residue 35 to improve IL-2 functional display. This effect was powerful enough to determine the isolation of super-secreted variants. Strikingly, mutations at position 35 did not emerge from CD25-mediated selection of yeast-displayed IL-2 variants^32^. This could reflect a lower influence of this residue in the yeast expression system or the insufficient sequence space coverage provided by rather small size yeast libraries, illustrating the value of mutagenesis scanning in different contexts.

Secretion of recombinant proteins is a complex sequence of events- including gene transcription, mRNA translation, protein folding and membrane transit. It is influenced by the gene sequence, the vector, the host cell, and the structural properties of the emerging polypeptide. K35E had similar effects on IL-2 secretion in two different contexts: periplasmic export and phage assembly in bacteria and secretion from mammalian cells. There were remarkable differences in the IL-2 DNA sequence itself (which was optimized for each host), the vectors (phagemid *versus* mammalian expression vector), the *N*-terminal signal sequences and the host cells. The only common element was the polypeptide sequence, pointing to a general advantage of the intrinsic changes induced by K35E in the structure/folding of IL-2. The impact of the initial amino acid stretch following the signal peptide on protein secretion has been well documented^37^. Although K35 is not located at this position, K35E is predicted to induce conformational changes in IL-2, particularly at its *N*-terminus. Increased flexibility and the specific *N*-terminal bend induced by the mutation could facilitate transit of the nascent polypeptide through cell membranes. The increased solubility of the molecule and its predicted higher stability are likely to favour proper folding and secretion by reducing the competing misfolding/aggregation processes that tend to reduce the yield and quality of recombinant proteins.

**Figure 8:**
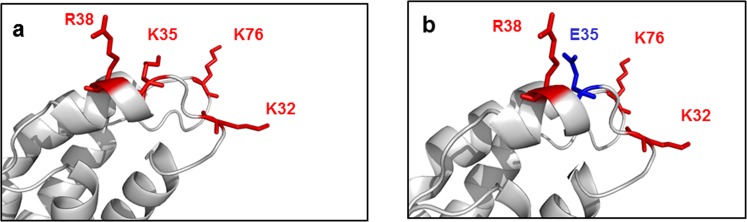
Charge distribution at the protruding nose end of native and K35E IL-2. Native IL-2 is represented in panel a, and its K35E mutated variant is shown in panel b. Protein backbones are represented as cartoons in grey. Charged residues at the protruding nose end of IL-2 cylinder are represented with coloured sticks in and labelled. Positively charged residues are in red, while the negatively charged Glu is blue.

The effects of K35E can be linked to the change in the local net charge. Ionic interactions between positively charged protrusions at the nose side of the IL-2 cylinder and negatively charged cavities at the tail have been postulated to mediate nose-tail docking of IL-2 molecules leading to the formation of polymeric aggregates^23^. K35E disrupts the charge distribution at the positive protruding end (K32, K35, R38, K76) and could thus affect aggregation (Fig. 8). The potential importance of disrupting this positive surface patch can go beyond specific structural features of IL-2, as there is a strong association of insolubility with the presence of positively charged surface patches among large sets of proteins^38^. Additionally, IL-2 has multiple hydrophobic segments classified as aggregation prone regions (APRs). Charged amino acids often flank APRs in highly expressed proteins and act as gatekeepers counteracting aggregation through repulsive forces^39^. Position 35 is adjacent to an APR (Supplementary Fig. S3). Although both the original K35 and the replacing Glu are charged residues (potential gatekeepers), their relative ability to protect from aggregation can differ. It has been reported that acidic residues are more hydrated than basic and other polar amino acids and that increasing the negative surface charge of proteins increases their solubility^40,41^.

The discovery of a simple structural solution (single mutation-based) reducing the remarkable aggregation propensity of IL-2 and improving its secretion contrasts with the failure of previous attempts to do so. Neither the introduction of *N*- and *C*-terminal fusions and artificial disulphide bonds nor the mutational disruption of the negatively charged cavity at tail produced the expected results, leading to the view that IL-2 aggregation propensity is too high to be conquered through engineering^23^. Changing the charge near the cleavage site between the signal peptide and the *N*-terminus of IL-2 (replacing K8/K9 by acidic residues) did not result in periplasmic secretion in *E. coli* host^42^. Our results illustrate the advantages of blind directed evolution to manipulate complex molecular interaction networks where the effects of individual modifications cannot be easily predicted. To our knowledge this is the first example of successful directed evolution of phage-displayed IL-2 to obtain novel molecules with valuable properties, after two decades of IL-2 phage display^27,28^ in a field dominated so far by yeast display^11,32^.

Phage display has been used to select modified proteins with higher secretion levels, particularly antibody fragments. The applications range from domain shuffling where full variable regions are replaced by new sequences determining improved secretion without affecting antigen recognition^43^, to mutagenesis scanning of the original sequence to optimize aggregation resistance and expression levels^44^. The current work resembles the latter case in the existence of a link between phage panning advantage and higher secretion from mammalian cells, the strong preponderance of negative residues among improved variants, and a highly positional effect of mutations. Whereas both examples differ from the massive introduction of charged amino acids in the so called ‘supercharged’ proteins^45^, our modification is even more subtle, as a single replacement works, in contrast to the requirement for two or more substitutions in the previous report^44^. Our selection strategy relied on an intrinsic periplasmic secretion advantage enhancing phage display, while pre-heating was used as an additional selection pressure on antibody variable domains^44^.

Despite the harmful effects of protein aggregation (loss-of-function and cell toxicity of aggregates) aggregation-prone regions are ubiquitous in all proteomes and some are evolutionarily conserved^46^. These regions, besides promoting non-physiological aggregation, stabilize the hydrophobic core of globular proteins, ensuring protein folding, and contribute to the formation of inter-molecular interfaces. This seems to be the case of IL-2, which forms extensive interfaces with its multi-chain receptor, including hydrophobic contacts^3,4^. While highly abundant proteins are less aggregation-prone, there is a subset of low abundance aggregation-prone proteins whose concentrations are tightly regulated, through slow transcription/translation and fast turnover, below critical levels that result in aggregate formation^47^. They are often involved in signal transduction and transcriptional activation during dynamic responses of cells to external perturbations^48^. Many immune-related cytokines, exhibiting an intrinsic aggregation propensity, share these properties, as they are secreted at very low levels in a highly regulated fashion, and are involved in cellular responses to immunological stimuli. Aggregation propensity might itself contribute to regulation, limiting the availability of soluble secreted molecules. This natural balance is disrupted in recombinant systems, where transcription/translation rates are maximized, folding-facilitating chaperones and quality control machinery are overloaded, and proteins greatly exceed their critical aggregation concentrations. That context determines the advantages of developing proteins that would probably never arise in nature, but are compatible with high level production in recombinant hosts. Mutated Interleukin-15 variants with increased solubility and expression levels^49^ illustrate the divergence between natural and directed cytokine evolution.

Extensive immunological characterization of the super-secreted IL-2-derived proteins is currently undergoing to assess their therapeutic value. The phage-based strategy to improve their biophysical properties and secretion could go far beyond this initial application, as IL-2 is a prototypic member of the four-alpha-helix bundle family of cytokines, sharing a common folding, immunological relevance and also manufacturability problems. Directed evolution on filamentous phages could thus give rise to a new wave of engineered cytokines showing not only the desired biological functions but also an enhanced developability potential.

## Methods

### Library construction, phage selection and characterization

Human IL-2 and its variants were displayed on filamentous phage as described^50^. Mutated versions and large synthetic libraries thereof were constructed by modified Kunkel mutagenesis^36,51^ on the original IL-2 gene template with mutagenic degenerate oligonucleotides. Phage rescue and selection were performed as described^51^, using immobilized CD25 (human/mouse) recombinant extracellular regions (R&D). Display levels and binding properties of IL-2 variants were determined by ELISA on polyvinyl chloride microplates coated with Myc1-9E10 mAb, the recombinant Fc-fused IL-2R extracellular regions, or anti-IL-2 mAbs^50^.

### Production and characterization of recombinant proteins

Expi293 cells (Thermo Fisher Scientific) were transiently transfected with constructs containing the synthetic genes coding for the extracellular domains of IL-2R subunits and for IL-2 variants, fused to the 5′ end of human Fc gene (cloned into the pCSE-2.6- hIgG1 Fc  vector)^52^. Cell transfection supernatants were evaluated in 12% SDS-PAGE. Recombinant proteins were purified by protein A-affinity chromatography. Binding properties of IL-2-containing fusion proteins (either purified or not) were evaluated by ELISA on polyvinyl chloride microplates coated with anti-IL-2 mAbs or the recombinant IL-2R subunits (R&D). Bound fusion proteins were detected with anti-human Fc antibodies conjugated to horseradish peroxidase (Sigma). The presence of soluble aggregates was analysed using a Prominence-i LC-2030C HPLC instrument (Shimadzu Europe GmbH) and a TSK gel G3000SWXL size exclusion column (TOSOH Biosciences).

### *In silico* analysis

GROMACS software package (version 4.6.5)^53^ was used for Energy Minimizations and MD simulations, using the AMBER99sb force field, and the TIP3P water model. The simulation systems consisted of free IL-2 (PDB code 2ERJ) or the mutated K35E IL-2 solvated in a truncated dodecahedral box with 27558 or 26378 water molecules, respectively. The MD simulations were sampled for 200 ns. The tripeptide systems GXG were similarly simulated. The folding free energy difference for the K35E variant with respect to the wt protein was calculated using the MM-PB(GB)SA method^54^. The tripeptide GXG (being X the amino acid of interest) was chosen as reference unfolded state^55^. The solvation free energy difference between IL-2 and the K35E variant was calculated using the polar solvation energy of the folded state obtained from the MM-GBSA calculations.

### *In vitro* determination of the biological activity of IL-2 and its variants

Purified Fc-fusion proteins containing IL-2 or its mutated counterparts were evaluated in the CTLL-2 proliferation assay as described^10^. Alamar blue dye reduction (the indicator of proliferation) was measured as the difference between Abs values at 540 and 630 nm. Doses inducing half-maximal proliferation were used to calculate the specific activity of each sample. The capacity of fusion proteins to stimulate CD8+ T lymphocytes was also evaluated *in vitro*. This population was isolated by flow cytometry from the spleens of C57BL/6 mice or from human peripheral blood mononuclear cells. The cells were labelled with CTV and stimulated with each protein during five days. All fluorochrome-conjugated mAbs used for flow cytometry were from eBioscience: For mouse samples, PE-conjugated anti-CD4 (L3T4), Pacific blue-conjugated anti-Foxp3 (NRRF-30), PECy7-conjugated anti-CD25 (3C7), PerCP Cy5.5-conjugated anti-ICOS, APCCy7-conjugated anti-CD8, FITC-conjugated anti-CD44 (IM7), PE-conjugated anti-CD122 (TMb1). For human samples, APCCy7-conjugated anti-CD8 (3B5) and BV605-conjugated anti-CD45RO (UCHL1). The numbers of proliferating cells were determined by flow cytometry on a LSR Fortessa flow cytometer (Becton Dickinson). Intracellular Foxp3 staining sets were purchased from eBioscience. Data were analysed using FlowJo software (TreeStar, Inc).

### *In vivo* analysis IL-2 function

Healthy C57/BL6 mice received five daily intraperitoneal injections of 4 × 10^4^ IU of K35E IL-2/Fc fusion protein or rhIL-2 or phosphate buffered saline (PBS) and were sacrificed on day 7. Spleens were collected, spleen weights were determined and cells were stained for flow cytometry analysis of CD8 + CD44^hi^CD122 + and CD4 + CD25 + Foxp3 + ICOS+ T cells. Samples were analysed as described above. To test the antimetastatic effect of the proteins, C57/BL6 mice were inoculated intravenously with 2 × 10^5^ cells from the melanoma MB16F0 cell line, followed by the above described treatments (with K35E IL-2/Fc, rhIL-2, or PBS). Mice were sacrificed on day 21 and lungs were collected to perform weight estimations. Data were analysed on the Graph Pad software. Parametric ANOVA and Bonferroni test for multiple comparisons were used for statistical analysis. Mice experiments were performed according to the guidelines of the International Laboratory Animals Research using standardized procedures at the Center of Molecular Immunology, Cuba and the at the Instituto de Medicina Molecular, Portugal. Food and water were administered *ad libitum*.

### Animal experiments’ statement

Protocols involving living animals at the Instituto de Medicina Molecular were approved by by ORBEA-iMM (the institutional Animal Welfare Body). Permission for animal experimentation was granted by DGAVG (Portuguese competent authority for animal protection). Institutional Committee of Animal Care and Use approved all the experimental protocols developed at the Center of Molecular Immunology. Animal experiments were performed in accordance with the relevant guidelines and regulations.

## Supplementary information

Suplemental information

## Data Availability

The data that support the findings of this study are available from the corresponding author upon reasonable request.
