# Supplementary material for: Directed evolution of super-secreted variants from phage-displayed human Interleukin-2
Source: Sci Rep. 2019 Jan 28;9:800. doi: 10.1038/s41598-018-37280-5 (PMC6349883; doi:10.1038/s41598-018-37280-5)
Supplement: Supplementary file 1 — Suplemental information [file 41598_2018_37280_MOESM1_ESM.pdf]

Supplemental information

Title:

Directed evolution of super-secreted variants from phage-displayed human Interleukin-2

Authors: Gertrudis Rojas[1*], Tania Carmenate[1], Julio Felipe Santo-Tomás[1], Pedro A. Valiente[2], Marlies Becker[3], Annia Pérez-Riverón[1], Yaima Tundidor[1], Yaquelín Ortiz[1], Jorge Fernandez de Cossio-Diaz[1], Luis Graça[4], Stefan Dübel[3], Kalet León[1]

Author affiliations:

[1]Center of Molecular Immunology, calle 216 esq 15, apartado 16040, Atabey, Playa, CP 11300, La Habana, Cuba

[2]Biology Faculty, La Habana University, 25 e/ I y J, Vedado, Plaza, CP 10400, La Habana, Cuba

[3]Technische Universität Braunschweig, Institute of Biochemistry, Biotechnology and Bioinformatics, Department of Biotechnology, Spielmannstraße 7, 38106 Braunschweig, Germany

[4]Instituto de Medicina Molecular, Faculdade de Medicina da Universidade de Lisboa, Lisbon, Portugal

*Correspondence should be addressed to G.R. (grojas@cim.sld.cu)

Supplementary Table S1. hIL-2-based library design

Position	Original residue in hIL-2	Amino acids (aa) in the library	Common properties
35	K	K, R	positively charged
38	R	K, R	positively charged
41	T	mixture of the 20 aa	-
42	F	F, I, L, V	hydrophobic
43	K	K, R	positively charged
44	F	F, I, L, V	hydrophobic
45	Y	F, Y	aromatic
61	E	E, D	negatively charged
62	E	E, D	negatively charged
64	K	mixture of the 20 aa	-
65	P	P, A	-
68	E	mixture of the 20 aa	-
69	V	mixture of the 20 aa	-
71	N	mixture of the 20 aa	-
72	L	L, M, V	hydrophobic
74	Q	mixture of the 20 aa	-
107	Y	mixture of the 20 aa	-

Library was constructed by Kunkel mutagenesis on the hIL-2 template gene cloned in the phagemid pCSM. Three mutagenic oligonucleotides were used to introduce diversity at the targeted positions (covering the segments K35-Y45, E61-Q74 and Y107). NNK codons coded for the mixture of the 20 amino acids in the library, whereas degenerate codons with lower diversity were used to code for pre-defined aa mixtures at some positions known to be functionally important for receptor binding.

Supplementary Figure S1. Reactivity of phage mixtures selected on immobilized CD25

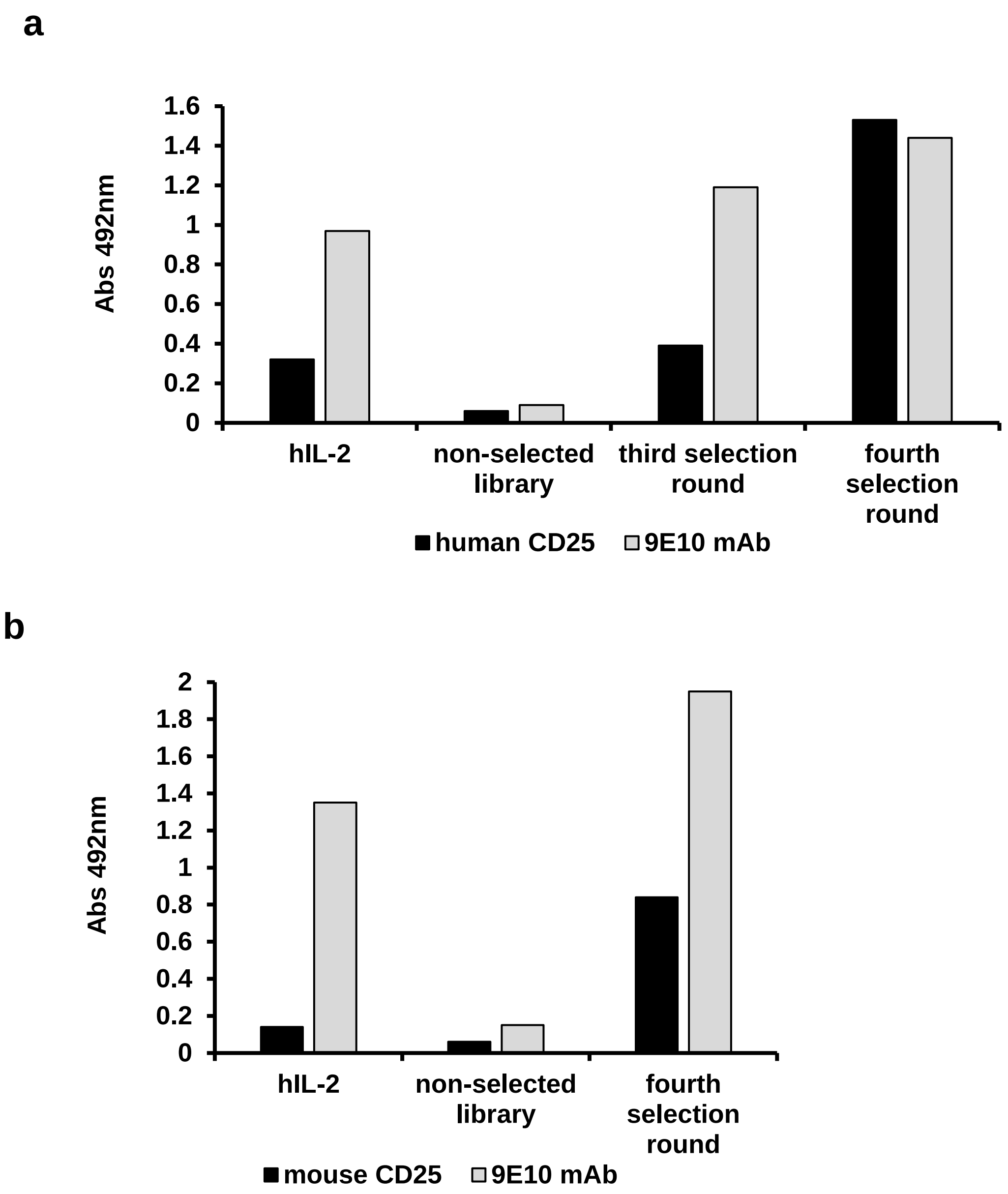

Reactivity of different phage pools (purified from the non-selected library and after several selection rounds) was evaluated by ELISA. Plates were coated with the selector molecule: CD25 of human (a) and mouse (b) origin and with the anti-*c-myc* tag Myc1-9E10 antibody. The latter was useful to show the differences between the display levels of phage pools. A phage pool displaying non-mutated hIL-2 was used as reference. Bound phages were detected with an anti-M13 mAb conjugated to horseradish peroxidase.

Supplementary Figure S2. Thermodynamic cycle used for folding free energy calculations

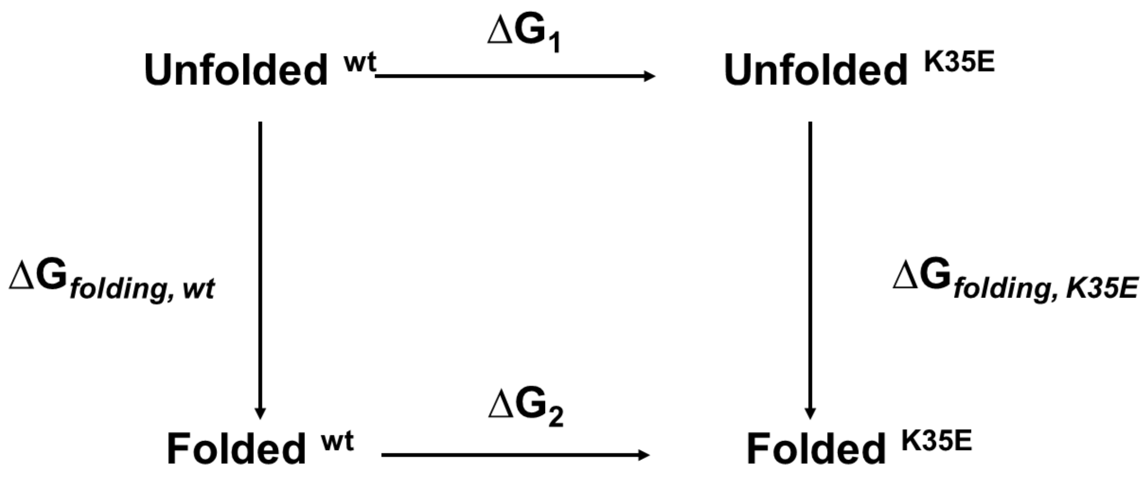

The folding free energy difference for the mutated K35E variant with respect to wild-type IL-2 protein was calculated using the MM-PB(GB)SA method. The tripeptide GXG was chosen as reference state of the unfolded polypeptide, where X is the amino acid of interest. From the thermodynamic cycle, the folding free energy difference between the wild type and mutated forms was calculated ($\Delta\Delta G_{folding} = \Delta G_1 - \Delta G_2$). A positive value of 17.49 kcal/mol was obtained, implying that K35E behaves as a stabilizing mutation.

Supplementary Figure S3. Location of aggregation-prone regions in human IL-2

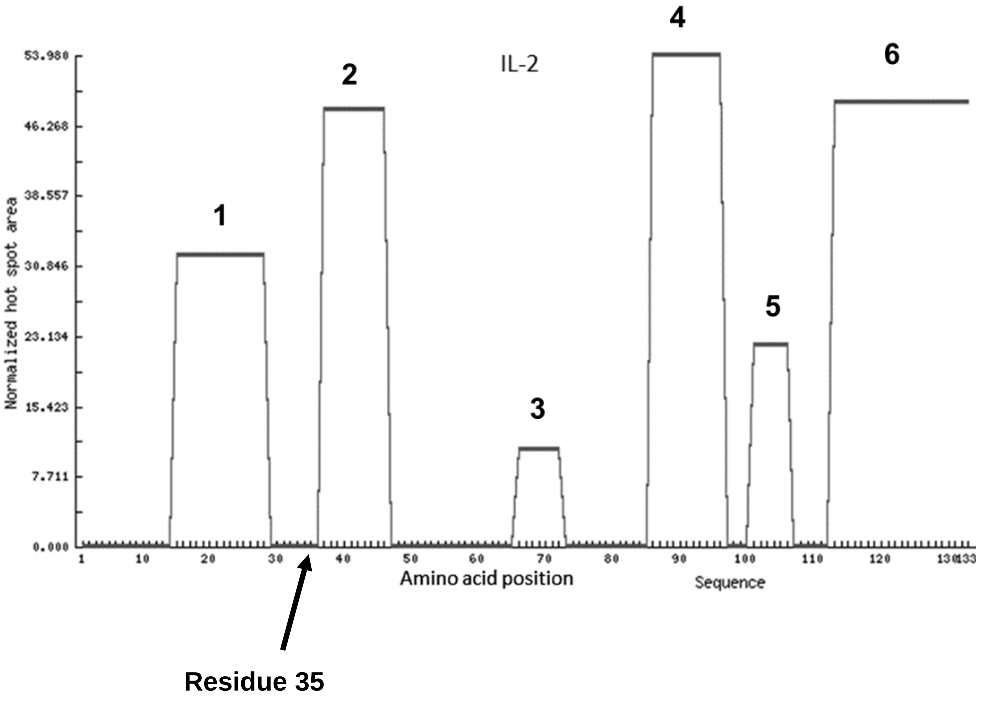

Six aggregation hot spots along the primary sequence of human IL-2 (1-6) were predicted *in silico* using AGGRESCAN algorithm. The second hot spot is adjacent to position 35 (indicated by the arrow).
